# Proteogenomics data for deciphering *Frankia coriariae* interactions with root exudates from three host plants^☆^

**DOI:** 10.1016/j.dib.2017.07.009

**Published:** 2017-07-11

**Authors:** Guylaine Miotello, Amir Ktari, Abdellatif Gueddou, Imen Nouioui, Faten Ghodhbane-Gtari, Jean Armengaud, Maher Gtari

**Affiliations:** a CEA, DRF/JOLIOT/DMTS/SPI/Li2D, Lab Innovative Technologies for Detection and Diagnostic, Bagnols-sur-Cèze, France; b Laboratoire Microorganismes et Biomolécules Actives, Université de Tunis El Manar (FST) & Université de Carthage (INSAT), 2092 Tunis, Tunisia

**Keywords:** Proteogenomics, Bacteria, Host plants, Interactions, Tandem mass spectrometry

## Abstract

*Frankia coriariae* BMG5.1 cells were incubated with root exudates derived from compatible (*Coriaria myrtifolia*), incompatible (*Alnus glutinosa*) and non-actinorhizal (*Cucumis melo*) host plants. Bacteria cells and their exoproteomes were analyzed by high-throughput proteomics using a Q-Exactive HF high resolution tandem mass spectrometer incorporating an ultra-high-field orbitrap analyzer. MS/MS spectra were assigned with two protein sequence databases derived from the closely-related genomes from strains BMG5.1 andDg1, the *Frankia* symbiont of *Datisca glomerata*. The tandem mass spectrometry data accompanying the manuscript describing the database searches and comparative analysis (Ktari et al., 2017, doi.org/10.3389/fmicb.2017.00720) [1] have been deposited to the ProteomeXchange with identifiers PXD005979 (whole cell proteomes) and PXD005980 (exoproteome data).

**Specifications Table**

**Table:** 

Subject area	*Environmental microbiology*
More specific subject area	*Frankia comparative proteogenomics*
Type of data	*Mass spectrometry raw files, Excel tables*
How data was acquired	*Data-dependent acquisition of tandem mass spectra using a Q-Exactive HF tandem mass spectrometer (Thermo).*
Data format	*Raw and processed*
Experimental factors	*Cells were incubated with filter sterilized root exudates derived from either compatible (Coriaria myrtifolia), incompatible (Alnus glutinosa) and non-actinorhizal (Cucumis melo) host plants, or without for the control.For each condition, three biological replicates were performed. From each condition, cells and supernatants (exoproteomes) were obtained by centrifugation.*
Experimental features	*The 12 cellular proteomes and 12 exoproteomes were briefly run on SDS-PAGE, followed by trypsin proteolysis. Tryptic peptides were analyzed by nano LC-MS/MS and spectra were assigned with the genome-derived protein sequence databases from strains BMG5.1 and Dg1.*
Data source location	*CEA-Marcoule, DRF-Li2D, Laboratory “Innovative technologies for Detection and Diagnostics”, BP 17171, F-30200 Bagnols-sur-Cèze, France*
Data accessibility	*Data is within this article and deposited to the ProteomeXchange via the PRIDE repository with identifiers PRIDE:*PXD005979*(whole cell proteomes) and*PXD005980*(exoproteome data).*

**Value of the data**

• The proteogenomics data are an invaluable resource for understanding *Frankia*/host plant interactions.
• A better coverage of *Frankia coriariae* BMG5.1 proteome is achieved by means of querying two closely-related genomes.
• The data have been exploited to decipher the main proteome changes in response to various root exudates. As described in detail in the accompanying manuscript [1], the proteins which are solely induced by *Coriaria myrtifolia* root exudates are involved in cell wall remodeling, signal transduction and host signals processing.

## 1 Experimental design and data

Interpreted tandem mass spectrometry results were acquired with a Q-Exactive HF instrument incorporating an ultra-high-field orbitrap analyzer. This mass spectrometer allows a rapid and deep coverage of proteome samples [2], [3], [4]. The results of peptide-to-spectra assignation were formatted in four.xls tables using the Microsoft excel program. The whole-cell proteome and exoproteome data from the 12 independent conditions were assigned to tryptic peptides against either the *Frankia BMG5.1* annotated genome or the *Frankia Dg1* annotated genome using the MASCOT 2.3.02 search engine (Matrix Science), with standard parameters: maximum number of missed cleavages at 2, mass tolerances for the parent ion and the product ions at 5 ppm and 0.02 Da, respectively, carbamidomethylated cysteine residues as fixed modification, oxidized methionine residues and deamidation of asparagine and glutamine as variable modifications, selection of peptides of at least 7 amino acids. For this, peptide-to-spectrum matches with a score above their peptidic identity threshold were filtered at *p*<0.05.

The use of two databases allows an improved coverage of gene products circumventing some erroneous annotations [5]. Supplementary Tables S1 and S2 list the peptide-to-spectrum matches for whole-cell proteomes queried against the BMG5.1 and Dg1 databases, respectively. A total of 149,629 and 144,213 MS/MS spectra were assigned, respectively. A total of 18,344 MS/MS spectra were specifically assigned with the Dg1 database, highlighting the interest of pan-proteomics [6]. Supplementary Tables S3 and S4 list the peptide-to-spectrum matches with all the tandem mass spectrometry characteristics for the exoproteomes queried against the BMG5.1 and Dg1 databases, respectively. The deposited data correspond to the 24 raw files and the interpreted files.

## 2 Materials and methods

### 2.1 Preparation of *Frankia coriariae* BMG5.1 samples

*Frankia coriariae BMG5.1* cells were grown in BD-N medium supplemented with 2.5 mM pyruvate as a carbon source at 28 °C. After ten days of cultivation, cells were supplanted with an equal volume of root exudates from each plant species that was previously filter sterilized. The cells were incubated for five additional days as described [1]. Cells were harvested by centrifugation. Proteins from the resulting supernatants were precipitated by trichloroacetic acid (10% final, w/vol). Cell pellets and exoproteins were dissolved in lithium dodecyl sulfate β-mercaptoethanol protein gel sample buffer (Invitrogen) and incubated at 99 °C for 5 min. They were processed as indicated previously [7]. For statistical purpose three independent biological replicates were performed for each condition.

### 2.2 Protein extracts and tandem mass spectrometry

The 24 peptide mixtures were analyzed by high-resolution tandem mass spectrometry using a Q-Exactive HF mass spectrometer (Thermo) coupled to an UltiMate 3000 LC system (Dionex-LC Packings) in similar conditions as those previously described [8]. Peptide mixtures (10 μl) were loaded and desalted on-line on a reverse phase precolumn (Acclaim PepMap 100 C18) from LC Packings. Peptides were then resolved onto a reverse phase Acclaim PepMap 100 C18 column and injected into the Q-Exactive HF mass spectrometer. The Q-Exactive HF instrument was operated according to a Top20 data-dependent acquisition method as previously described [8], selecting 2+ and 3+ possible charge states.

### 2.3 Protein sequence database for proteogenomics MS/MS assignment

The recorded MS/MS spectra for the 12 whole-cell proteome samples and the 12 exoproteome samples were searched against the genome-derived protein sequence databases from *Frankia* strains BMG5.1 and Dg1 with standard parameters for microbial proteomics [9], [10], [11], [12].The number of MS/MS spectra per protein (spectral counts) was determined for the three replicates of each of the four conditions which were assayed.

## Appendix A Supplementary material

Table S1. List of peptide-to-spectrum assignments and their characteristics for whole-cell proteomes queried against BMG5.1 database. Supplementary material

.

Table S2. List of peptide-to-spectrum assignments and their characteristics for whole-cell proteomes queried against Dg1 database. Supplementary material

.

Table S3. List of peptide-to-spectrum assignments and their characteristics for exoproteomes queried against BMG5.1 database. Supplementary material

.

Table S4. List of peptide-to-spectrum assignments and their characteristics for exoproteomes queried against Dg1 database. Supplementary material
